# Determination of the miRNA profile of extracellular vesicles from equine mesenchymal stem cells after different treatments

**DOI:** 10.1186/s13287-025-04287-5

**Published:** 2025-04-05

**Authors:** Michele C. Klymiuk, Julia Speer, Isabelle De Marco, Mohamed I. Elashry, Manuela Heimann, Sabine Wenisch, Stefan Arnhold

**Affiliations:** 1 Institute of Veterinary-Anatomy, -Histology and -Embryology, Faculty of Veterinary Medicine, Justus-Liebig-University Giessen, Frankfurter Strasse 98, 35392 Giessen, Germany; 2 Clinic of Small Animals, c/o Institute of Veterinary-Anatomy, -Histology and -Embryology, Faculty of Veterinary Medicine, Justus-Liebig-University Giessen, Frankfurter Strasse 98, 35392 Giessen, Germany

**Keywords:** MSC, EVs, NGS, miRNA, Osteoarthritis, Damage model

## Abstract

**Background:**

Osteoarthritis (OA) is a common and incurable disease in humans and animals. To gain a better understanding of the pathogenesis and identify potential treatments, miRNAs will be extracted and analysed from extracellular vesicles (EVs) of equine adipose derived mesenchymal stem cells (AdMSCs).

**Methods:**

For this purpose we cultivated and pretreated AdMSCs under different conditions: interleukin 1β, shock wave, chondrogenic differentiation, chondrogenic differentiation under hypoxia, or after senescence. After treatment, EVs were harvested from the cell culture supernatants. Next-generation sequencing (NGS) was used to sequence the miRNAs from the EVs.

**Results:**

A total of 89 miRNAs whose expression was significantly altered compared with that of an untreated negative control were identified. On average, 53 miRNAs were upregulated and 6 miRNAs were downregulated. Among others, the miRNAs eca-miR-101, eca-miR-143, eca-miR-145, eca-miR-146a, eca-miR-27a, eca-miR-29b, eca-miR-93, eca-miR-98, and eca-miR-221 were significantly increased after the stimulations, which, as known anti-inflammatory miRNAs, could be candidates for therapeutic use in the treatment of OA.

**Conclusion:**

These results lay the foundation for further research into the significance and efficacy of these miRNAs so that this knowledge can be improved in further experiments and, ideally, translated into therapeutic use.

**Supplementary Information:**

The online version contains supplementary material available at 10.1186/s13287-025-04287-5.

## Background

Osteoarthrosis (OA), also known as osteoarthritis, is a disease that occurs more frequently, particularly in older individuals. This applies both in human medicine [1] and in veterinary medicine [2, 3]. It is considered an incurable, painful degenerative disease of the joint [4]. It is a very heterogeneous disease, accompanied by profound structural changes in the synovial membrane, the cartilage, and the subchondral bone of the joint [5]. For a long period of time, the most important part of treatment was providing patients with adequate analgesic therapy with nonsteroidal anti-inflammatory drugs [6, 7]. These are often combined with intra-articular corticosteroids, hyaluronic acid administration [8], or other adjuvant measures such as a change in diet or weight reduction [7]. Finally, the insertion of a joint prosthesis is often the method of choice to restore the function of the joint with the least possible pain, especially in human medicine [9]. Mesenchymal stem cells constitute another promising treatment method in this context [10], which in today’s context should be called mesenchymal stromal cells or medicinal signaling cells [11]. It has since been recognized that the function of MSCs is not to self-differentiate into appropriate tissues but rather a reaction in their local area, including a healing process through the coordination and stimulation of immune and tissue cells [11, 12].

In recent years, extracellular vesicles (EVs) in particular have emerged from the secretome of MSCs as special mediators [12, 13]. The use of MSC-derived EVs has several advantages over the transplantation of MSCs themselves. In addition to their significantly faster, standardizable production compared with the cultivation of autologous MSCs, they can also maintain consistent quality and reduce the risk of side effects when living cells are used [14]. Extracellular vesicles are vesicles released by nearly all cells. They are coated with a lipid double membrane and have a size in the nanometer range. EVs are released by cells into the extracellular space and can contain various substances, including proteins, lipids, and nucleic acids such as microRNA (miRNA) [15]. Supportive effects for the treatment of OA in general have already been demonstrated for EVs [16] as well as specific associations with miRNAs contained in EVs [17, 18].

This led us to investigate miRNAs in EVs, both from undifferentiated MSCs, as they could be used for the treatment of OA, and from chondrogenically differentiated MSCs, as a possible damage model for the lack of repair in joints affected by OA. Specifically, we want to find out what changes occur in the content of EVs when they are not kept under comparatively normal (cell culture) conditions, but instead are stimulated in different ways. This will involve either a damaging stimulation, such as treatment with interleukin 1β, or a more pro-cartinogenic stimulation in the form of chondrogenic differentiation of MSCs. The comparison of these two approaches should give us an indication of which changes in EVs, here regarding the content of miRNAs, result from these treatments and which potential miRNAs can be found that should be further investigated for a potential treatment of OA. We are using the equine model for this, both because we see a benefit for the care of this species, and because it is a suitable model for human OA.

## Methods

### AdMSC collection

The donors required for the experiments were already available in the cryobank at the required passages. Specifically, they came from horses in which adipose tissue was taken during a noninflammatory surgical procedure or directly after slaughter at the abattoir. The sampling procedure has already been published [19]. The samples were collected from three horses, a stallion, a gelding, and a mare. The average age was 13.3 (3–19) years. The authorization for this was obtained from the local authorities and is registered under the number V 54 − 19 c 20 15 h 02 GI 18/1 kTV 1/2018.

### AdMSC cultivation and differentiation

Equine adipose-derived MSCs were incubated at 37 °C and 5% CO_2_ in a humidified atmosphere. For this purpose, cryopreserved portions of AdMSCs at passage two were rapidly thawed in a 37 °C water bath and then incubated with standard medium consisting of DMEM low glucose (DMEM-LG, order no. 11564446, Thermo Fisher Scientific, Germany), 10% fetal calf serum (FCS, batch no 201004, PAN, Germany) and 1% antibiotics (Penicillin-Streptomycin, order no. 11548876, Fisher Scientific, Germany) by centrifugation at 300 × g for 5 min, after which the cryomedium was discarded. The cells were then placed in 75 cm² cell culture flasks (order no. 734–2066, VWR, Germany) at a concentration of 0.5 million cells per bottle with 12 ml of standard medium. When the cells reached approximately 80% confluence, they were passaged again for the experiment, so that they were at passage 3.

The detection of mesenchymal stem cells is routinely performed in our laboratory following the International Society for Cellular Therapy (ISCT) standards [20] and is not described in detail here. Briefly, cells obtained from various donors, were examined for the following characteristics of stem cells: plastic adherence, differentiation into three typical lineages (osteogenic, adipogenic, and chondrogenic), and flow cytometric validation of stem cell-specific markers, such as CD90 (positive marker, monoclonal antibody clone 5E10), CD44 (positive marker, monoclonal antibody clone IM7), MHC II (negative marker, monoclonal antibody clone CVS20) and CD 45 (negative marker, monoclonal antibody clone UCHL1) [21, 22, 23].

The differentiations into the osteogenic and adipogenic lineages were carried out in 24-well plates (order no. 734–2325, VWR, Germany) for 2 weeks in the respective osteogenic media (DMEM-LG, 5% FCS, 1% antibiotics, 0.1 µM dexamethasone, 10 mM beta-glycerolphosphate, 60 µM ascorbic acid) or adipogenic media (DMEM low glucose, 5% FCS, 1% antibiotics, 1% insulin-transferrin-selenite solution (ITS, order no. ITS-H, Capricorn, Germany), 1 µM dexamethasone, 2.5 µM rosiglitazone). Chondrogenic differentiation was carried out in an antiadhesive 96-well plate with a U-shaped bottom (order no. 734–2782, VWR, Germany) in a pellet culture for 2 weeks. A medium of DMEM-LG, 1% antibiotics, 1% ITS, 0.1 µM dexamethasone, 0.9 mM sodium pyruvate, 0.17 mM ascorbic acid, 0.35 mM proline, and 10 ng/ml TGFb was used to induce chondrogenic differentiation.

After the incubation period, alizarin-red staining was used to detect osteogenic differentiation, oil red O staining was used to detect adipogenic differentiation and alcian-blue staining was used to detect chondrogenic differentiation after fixation and sectioning of the pellets [23].

### AdMSC treatment

#### Chondrogenic differentiation

Chondrogenic differentiation was performed in a monolayer on cell culture plates with d = 10 cm (order no. 734–2321, VWR, Germany). The cells were incubated for 21 days with the chondrogenic differentiation medium (DMEM-LG, 1% antibiotics, 1% ITS, 0.1 µM dexamethasone, 0.9 mM sodium pyruvate, 0.17 mM ascorbic acid, 0.35 mM proline, 10 ng/ml TGFb).

#### Chondrogenic differentiation under hypoxia

In addition, chondrogenic differentiation was performed in a monolayer under hypoxia at 3% O_2_ as previously described.

#### Interleukin 1β treatment

For treatment with interleukin 1β (IL-1β, order no. 200-01B, Thermo Fisher Scientific, Germany), AdMSCs at approximately 80% confluence were incubated in a 75 cm² cell culture flask in DMEM LG, 1% P/S and 1% ITS for 3 days. For the actual treatment, 10 µg/ml IL-1β (manufacturer) was separately added to the cell culture medium every day.

#### Shockwave treatment

**Fig. 1:**
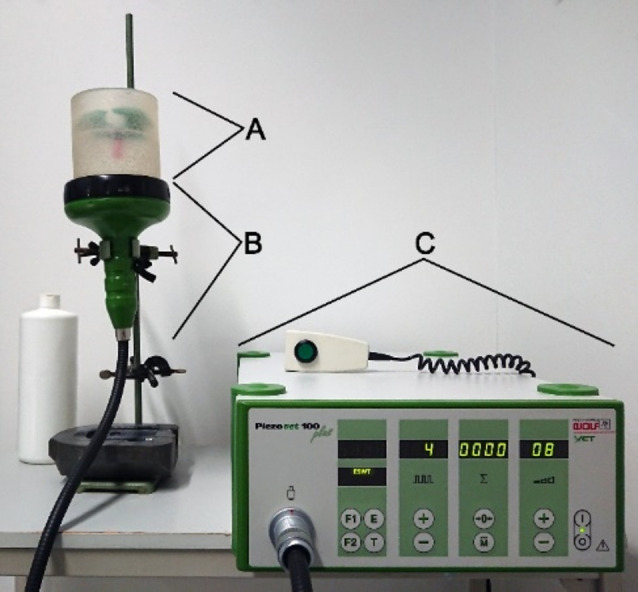
Illustration of the experimental setup for the shock wave treatment of AdMSCs. The silicone cup with the sample tube (marked with **A**, see details in Fig. 2), the transducer (marked with **B**) and the shock wave generator (marked with **C**)

**Fig. 2:**
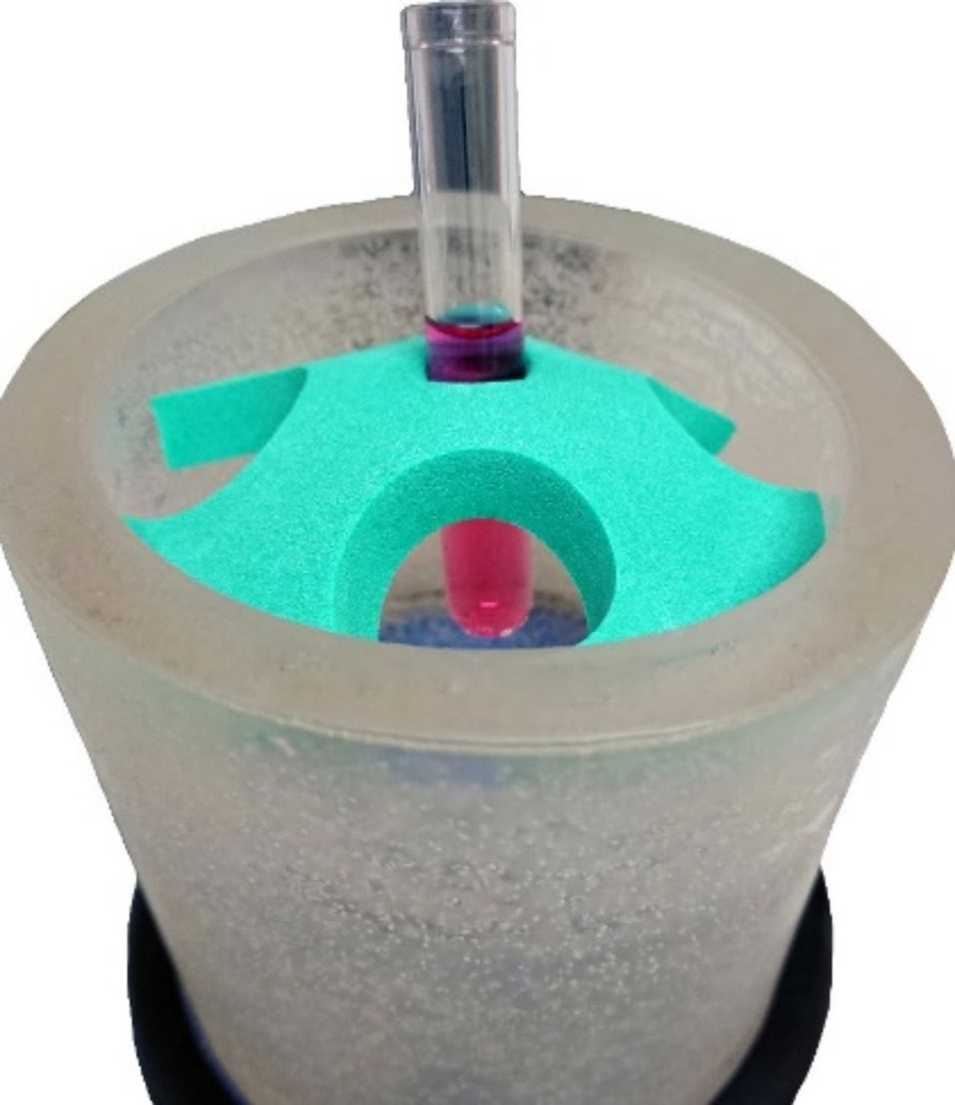
Detailed enlargement of Fig. 1, A. The silicone cup, which is filled with water for sound wave transmission, is colored gray. The sample tube with the cell suspension (red liquid) was immersed in it. The tube is fixed in place with a green foam holder. For better visualization, the tube was pulled slightly upward from the water

Treatment with shock waves was performed with an extracorporeal shock wave device (Piezovet 100 plus, Richard Wolf GmbH, Germany) and the FB12 G5 transducer (Richard Wolf GmbH, Germany). In a focal volume of 59 mm², an energy flux density of 1.1 mJ/mm² and a pressure of 122 MPa is applied, which corresponds to the maximum treatment level of “20”. The experimental setup is shown in the Figs. 1 and 2.

Since the cells for this treatment must be present in a cell suspension to concentrate them in the focus volume, the cells obtained after passage 2 were first adjusted to a concentration of 1 × 10^6^ cells/ml. Then, 9 ml of this suspension (containing 9 million cells) was dispensed into three 5 ml polypropylene tubes (order no. 55.526.006, Sarstedt, Germany) with 3 ml per tube. The tubes were placed in a custom silicone cup filled with water at the focus of the shock waves. The tubes were then sequentially exposed to 1000 pulses at a frequency of 8 Hz at the maximum level of the device (level 20). The AdMSCs were then recultured together in a 75 cm² cell culture flask in standard medium for 8 h. Afterwards, the FCS-containing medium was removed by washing with a PBS solution, and a further incubation period of two days followed by standard medium without FCS, supplemented with 1% ITS instead.

#### Induction of senescence

For the induction of senescence, the AdMSCs were further cultured until passage 20 when they reached their Hayflick limit. At passage 20, the AdMSCs were switched to FCS-free medium supplemented with 1% ITS at approximately 60% confluence and cultured for another 2 days.

In addition, senescence in a 6-well plate (order no. 734–2323, VWR, Germany) with confluent cells was confirmed after staining for beta-galactosidase. For this purpose, AdMSCs were fixed in 6-well plates with 4% paraformaldehyde, washed with PBS and then incubated overnight at 37 °C with beta-galactosidase staining solution. This staining solution consisted of a PBS solution with 150 mM NaCl, 1 mg/ml x-GAL, 5 mM K-hexacyanoferrate II, 5 mM K-hexacyanoferrate III, 2 mM MgCl_2,_ and 40 mM citric acid. After staining, the excess dye was again rinsed off with PBS.

### Isolation and collection of EVs

After the AdMSCs were cultured in serum-free medium, EVs were harvested from the cell culture supernatant. For this purpose, the EV-containing supernatant was removed from the cell culture flasks, which corresponded to a volume of 15 ml per 75 cm² flask. Since three 75 cm² flasks were used to obtain a minimum amount of EVs, a total of 45 ml of medium was obtained for each type of pretreatment (standard medium, hypoxia, IL-1β, shock wave, chondrogenic differentiation under normoxia and hypoxia) and transferred to a 50 ml centrifuge tube. These samples were centrifuged for 5 min at 2,700 × g to remove cell debris, and the supernatant was removed again and filtered through a 0.2 μm syringe filter (order no. 83.1826.001, Sarstedt, Germany).

### Nanoparticle analysis and statistics

For quality assurance, 3 ml of this filtrate was then measured via a nanoparticle tracking analysis (NTA) device to ensure that the measured samples actually corresponded to the EVs. This measurement was performed via a NanoSight LM10 (Malvern Instruments Ltd., UK) and NTA 3.3 software (Malvern Instruments Ltd., UK) and is described in detail elsewhere [24]. Characterization by transmission electron microscopy (TEM) in conjunction with immunogold labeling for the tetraspanins CD9 (monoclonal antibody clone HI9a) and CD81 (monoclonal antibody clone sc-166,029) as well as western blotting of the tetraspanin CD9, was performed previously [24]. The differences in the size and number of nanoparticles obtained were analyzed for significance via one-way analysis of variance (ANOVA) with repeated measurements. If there were significant group differences, a Tukey test was then carried out to identify the significantly altered condition.

### NGS analysis

A 20 ml volume was taken from the remaining supernatant and stored separately at -80 °C until the samples were collected and sent to a commercial provider for next-generation sequencing to detect the contained miRNA. For the detection of miRNAs a “TrueQuant SmallRNA Seq Kit” was used according to the manufacturer’s instructions (GenXPro GmbH, Germany). The method employs a gel-free, single-tube protocol for high-yield small RNA NGS library generation. Sequencing was performed on an Illumina NextSeq500 system via 1 × 75 sequencing cycles.

### NGS data processing

Unprocessed sequencing reads were adapter-trimmed and quality-trimmed via Cutadapt (version 4.6 [25]), with the arguments “-e 0.1 -O 3 -q 20 -m 14 -n 8”. FastQC (0.11.9 [26]), was used to assess the quality of the sequencing reads. Processed sequencing reads were mapped via Bowtie2 (2.4.4 [27]), on trna (with arguments: “--sensitive --local”) and mirna (with arguments: “--local --ma 1 --score-min L,0,0.9 --mp 1,1 --rdg 2,1 --rfg 2,1”) and ENSEMBL_cdna (with arguments: “--sensitive --local”) and ENSEMBL_ncrna (with arguments: “--sensitive --local”) of EquCab3.0 (*Equus caballus*). The mapping was performed iteratively, meaning that only those reads not mapping to the previous reference were mapped to the next one. The Quantification of the reads mapped to each transcript was performed via HTSeq (version 2.0.2 [28]), trna (with arguments: “-i transcript_id -r name -a 0 -m union” and strandedness “no”) and mirna (with arguments: “-I transcript_id -r name -a 0 -m union” and strandedness “no”) and ENSEMBL_cdna (with arguments: “-i transcript_id -r name -a 0 -m union” and strandedness “no”) and ENSEMBL_ncrna (with arguments: “-i transcript_id -r name -a 0 -m union” and strandedness “no”). MultiQC (version 1.25.1 [29]), was used to create a single report that visualizes output from multiple tools across many samples, enabling global trends and biases to be quickly identified.

### Differential expression analysis (DEA), statistics

The DEA was performed with DESeq2 (version 1.38 [30]), . Only entries that had a raw value of at least 5 in at least 2 samples were used in the DEA. The Log2FoldChange values were shrunk with “ashr” [31]. The DEA results with an FDR-adjusted p-value less than or equal to 0.05 and an absolute log2FoldChange greater than or equal to 1 were labeled as significant via the Wald test. Correction of p values after multiple testing was performed via the Benjamini-Hochberg method.

### Heatmap generation

A heatmap was created via an online tool [30] to provide a clear representation of the changes in miRNA expression. The average linkage method was selected as the clustering method and Euclid was selected as the distance measurement method.

## Results

### Cultivation and characterization of equine adipose derived MSCs

**Fig. 3:**
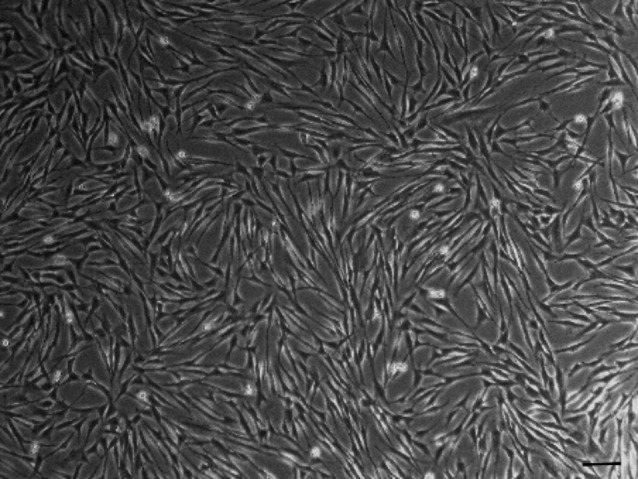
Cultivated AdMSCs adhere to the plastic bottom of the cell culture flask. The scale bar corresponds to 100 μm

**Fig. 4:**
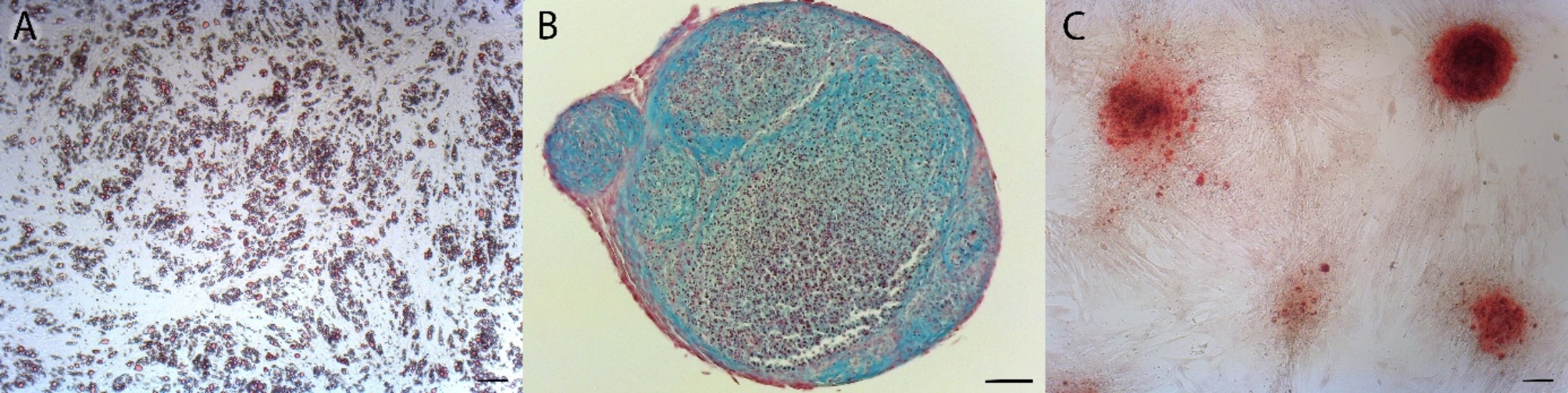
The three directions of AdMSC differentiation are shown. **A**) Intracellular staining of fat vacuoles with Oil Red O. **B**) Histological section through a 3D chondro-pellet after chondrogenic differentiation. The sulfated proteoglycans were stained with Alcian blue. **C**) Distinct nodule formation after osteogenic differentiation with calcium accumulation clearly stained red by Alizarin staining. The scale bar corresponds to 100 μm

**Fig. 5:**
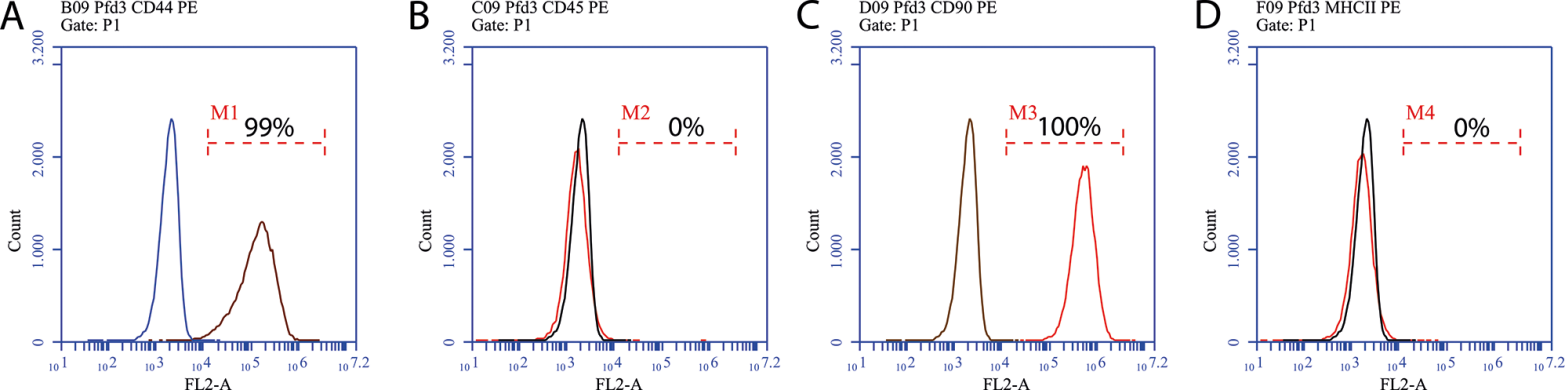
Flow cytometric characterization of AdMSCs. The surface markers CD44 (**A**) and CD90 (**C**) were detected as positive, and CD45 (**B**) and MHC II (**D**) were negative

As defined for MSCs, the AdMSCs used, exhibited clear plastic adherence to the cell culture flasks (Fig. 3). Differentiation into the adipogenic, osteogenic and chondrogenic lineages was also demonstrated (Fig. 4). According to general MSC characteristics flow cytometric analysis revealed the expression of surface markers such as CD44 and CD90 and the absence of a CD45 and MHC II expression (Fig. 5).

### Proof of senescence

**Fig. 6:**
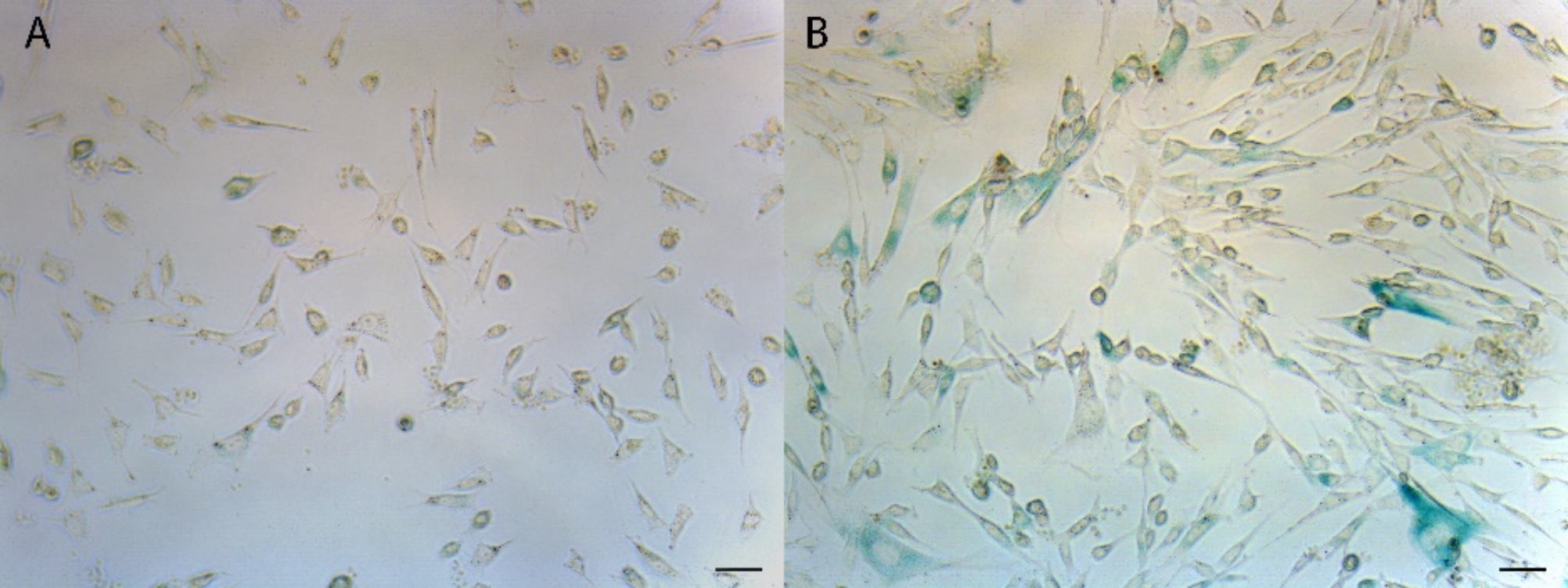
Equine AdMSCs at passage 5 (**A**) and passage 20 (**B**) after beta-galactosidase staining for the detection of senescent cells. Positive light blue coloration is clearly visible at passage 20. The scale bar indicates 50 μm

Compared with those at lower passages, positive beta-galactosidase detection in AdMSCs at passage 20, as detected by clear bluish coloration, demonstrated the senescence characteristic of stem cells (Fig. 6).

### NTA analysis of the media supernatant

**Fig. 7:**
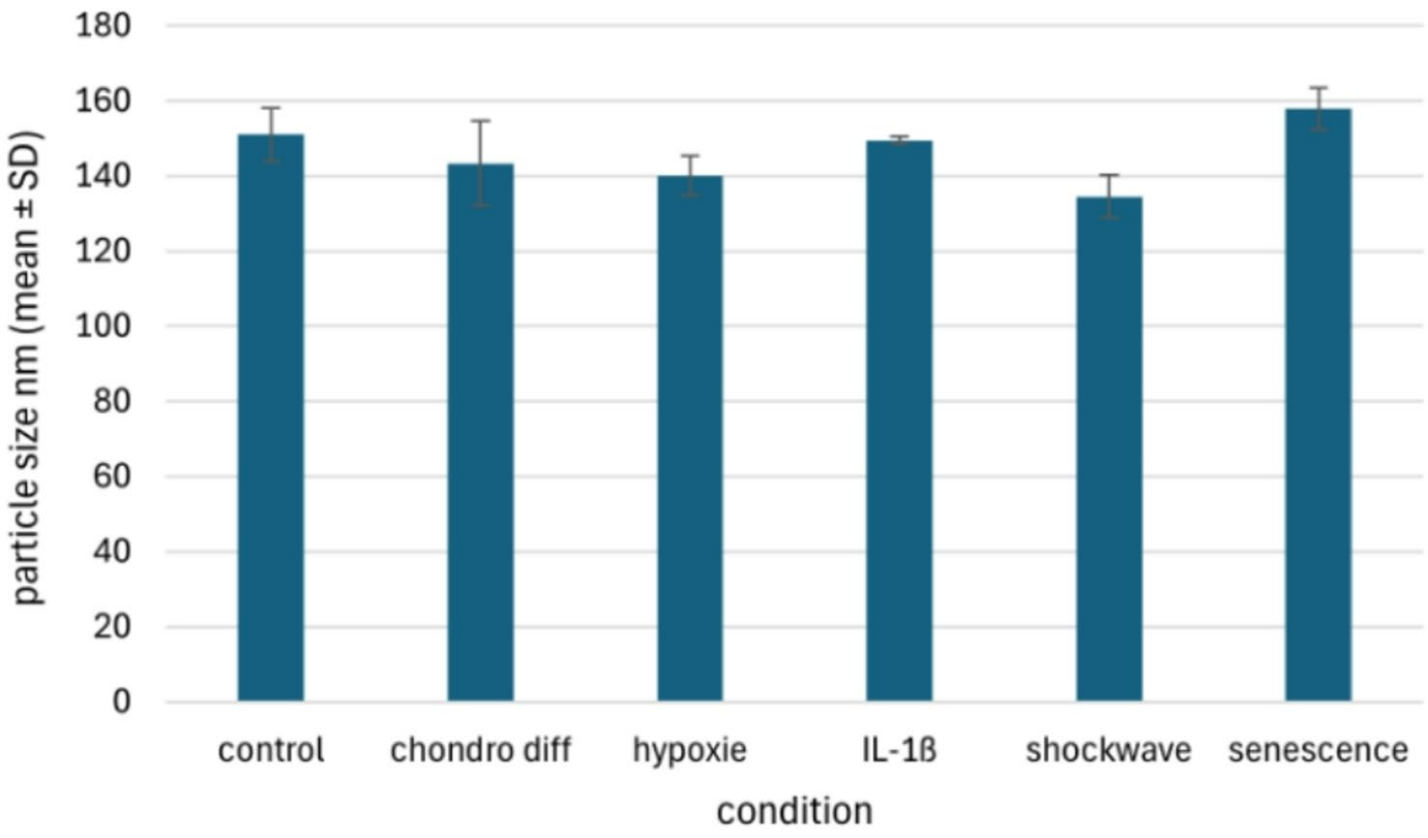
Particle size of EVs in the supernatant of cultured equine AdMSCs after the different treatments. The different conditions previously described are as follows: negative control without prior treatment (control), chondrogenic differentiation (chondro diff), chondrogenic differentiation under hypoxia (hypoxia), treatment with IL-1β, shockwave treatment (shockwave) and senescent AdMSCs (senescence)

**Fig. 8:**
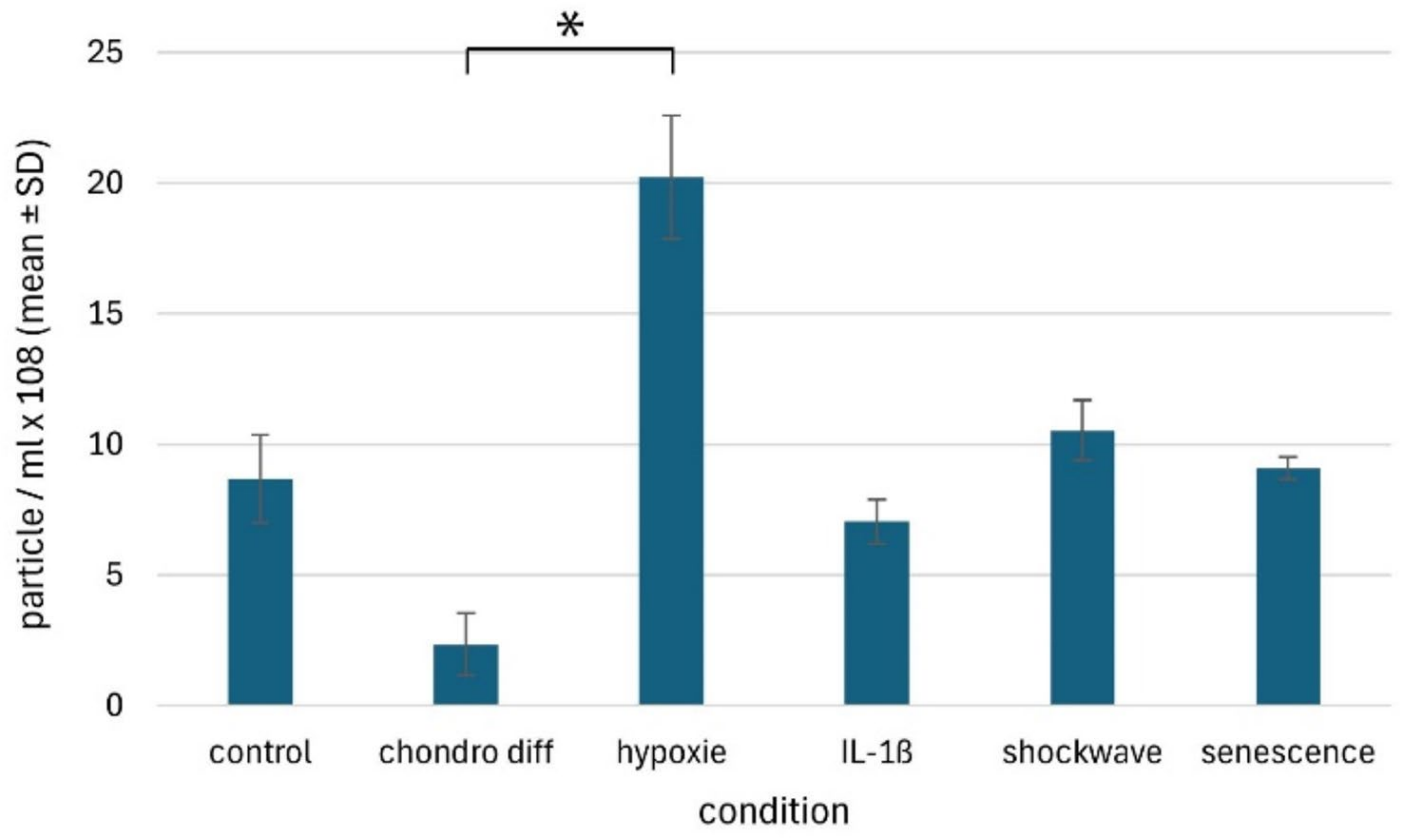
EV concentrations in the supernatants of cultured equine AdMSCs after different treatments. The different conditions previously described are as follows: negative control without prior treatment (control), chondrogenic differentiation (chondro diff), chondrogenic differentiation under hypoxia (hypoxia), treatment with IL-1β, shockwave treatment (shockwave) and senescent AdMSCs (senescence). The asterisk indicates a significant difference (*p* = 0.029)

After the cell culture supernatants enriched with EVs were obtained, the size and number of nanoparticles contained in the supernatants were measured via NTA. Since no clear distinction can be made between EVs and other cellular or other components, the nanoparticles contained in the medium, which also contain the EVs, must be measured at this point. The average size under all conditions (negative control, IL-1β stimulation, chondrogenic differentiation with and without hypoxia, shock wave treatment, and senescence) was relatively the same with a size of 146.01 ± 7.67 nm (x̅ ± SD, Fig. 7). There were no significant differences between the different treatment options (*p* = 0.075). On the other hand, there were differences in the number of nanoparticles obtained (Fig. 8). Here, the average concentration was 9.66 ± 5.39 × 10^8^ particles/ml (x̅ ± SD). A lower concentration of nanoparticles after chondrogenic differentiation with 2.35 ± 1.19 particles/ml (x̅ ± SD) was particularly evident, compared to a significantly increased concentration after chondrogenic differentiation under hypoxia. Here, 20.23 ± 2.36 particles/ml (x̅ ± SD) were present in the cell culture supernatant. A significant difference of *p* = 0.029 was found between these two concentrations. In general, however, there were no significant differences between the various treatments and the negative control, i.e. untreated AdMSC, at a concentration of 8.70 ± 1.67 × 10^8^ particles/ml (x̅ ± SD).

**Fig. 9:**
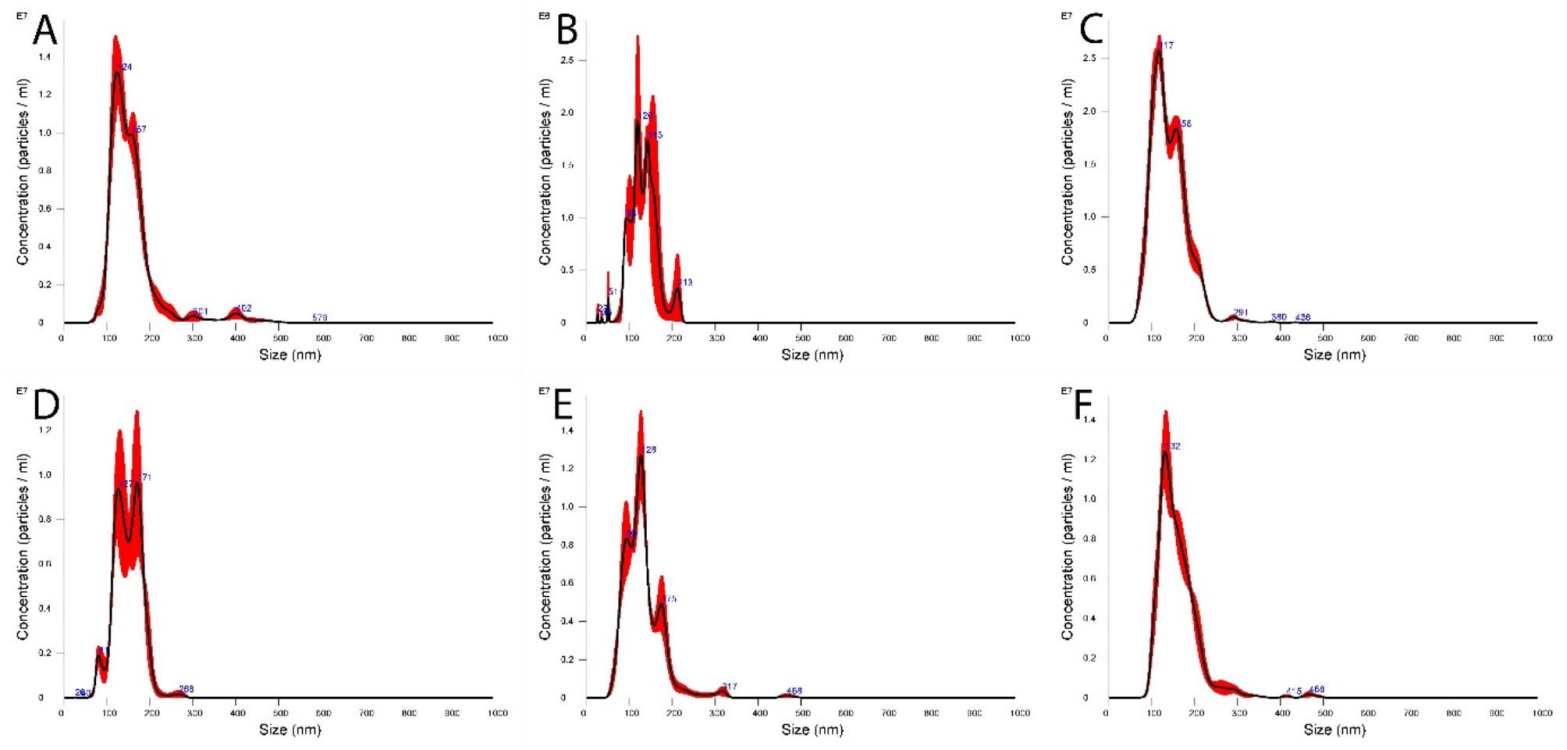
Particle size distributions are visualized after analysis via a nanoparticle tracking analysis (NTA) instrument. Measurements were carried out after different pretreatments: negative control without prior treatment (**A**), chondrogenic differentiation (**B**), chondrogenic differentiation under hypoxia (**C**), treatment with IL-1β (**D**), shockwave treatment (**E**) and senescent AdMSCs (**F**)

Compared with the respective treatment methods, the size of the respective nanoparticles was no longer extremely homogeneous. This is shown in Fig. 9.

### NGS anaylsis

**Fig. 10:**
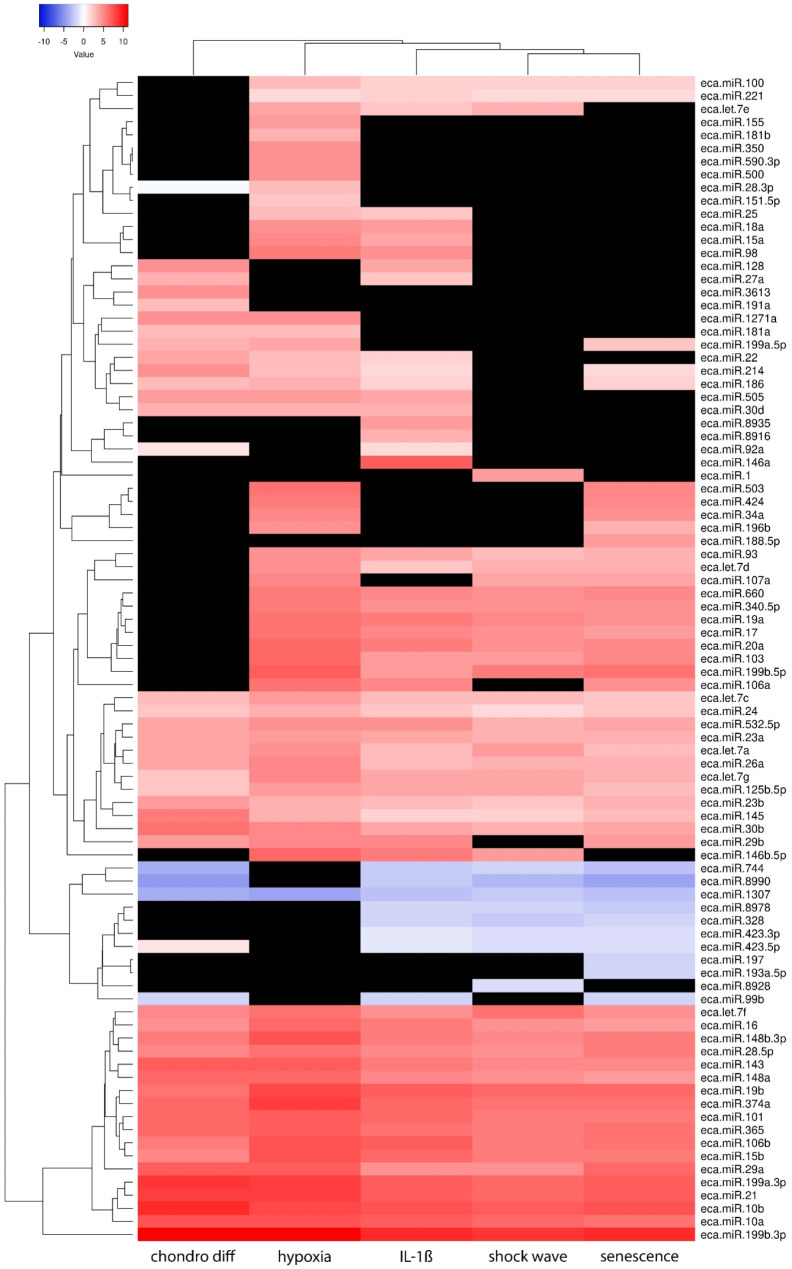
Heatmap of significantly altered miRNA expressions after different treatments of equine AdMSCs. These were chondrogenic differentiation (chondro diff), stimulation with interleukin 1β (IL-1ß), shockwave treatment (shockwave), chondrogenic differentiation under hypoxia (hypoxia) and senescence in AdMSCs (senescence)

**Fig. 11:**
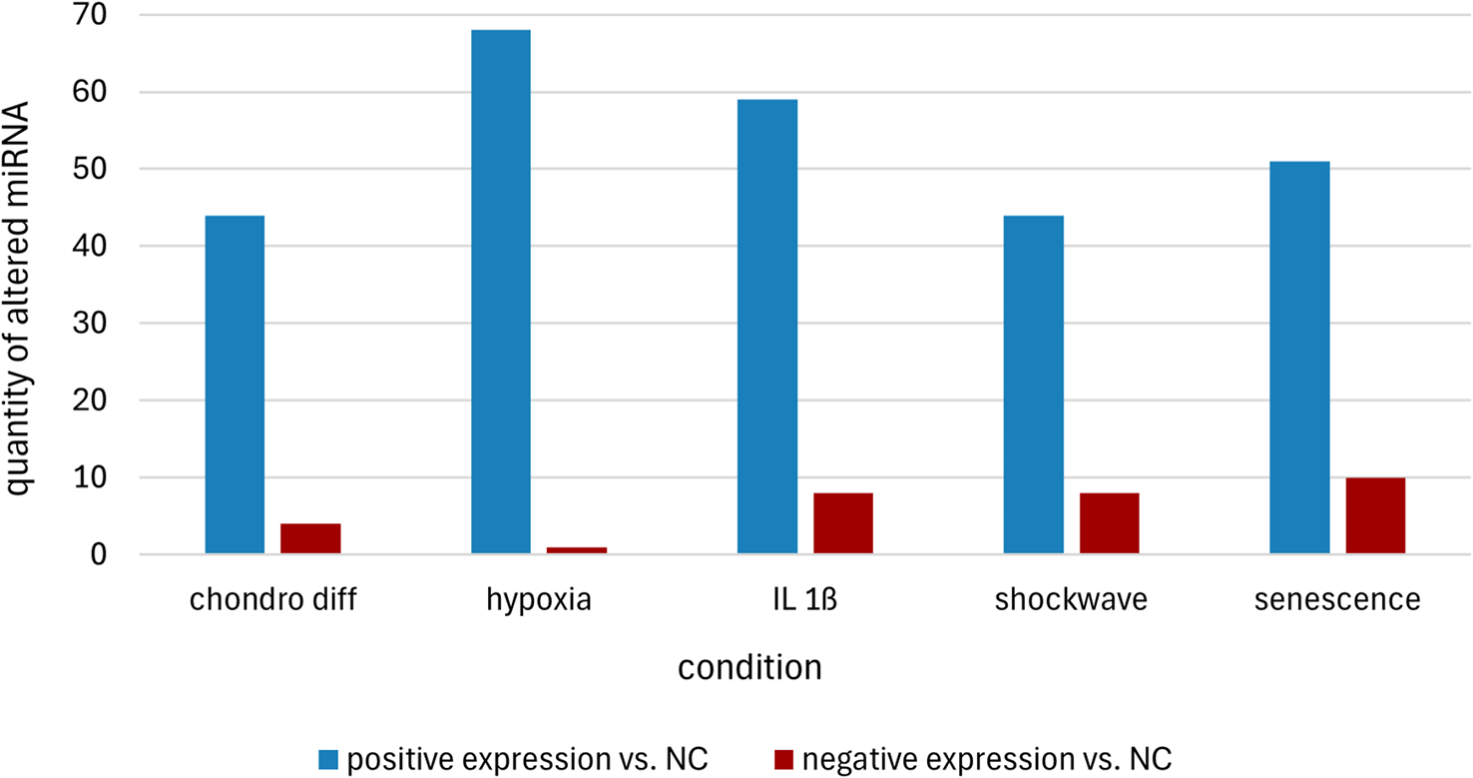
Representation of the number of significantly changed miRNA expressions after different treatments of equine AdMSCs. The sums of the significantly changed miRNA expressions compared with the negative control (NC) are shown. The various conditions previously described are as follows: negative control without prior treatment (control), chondrogenic differentiation (chondro diff), chondrogenic differentiation under hypoxia (hypoxia), treatment with IL-1β (IL 1ß), shockwave treatment (shockwave) and senescent AdMSCs (senescence)

After next-generation sequencing and raw data processing, the average sequencing depth per sample was 4,872,746 reads. On average, 51.04% of the reads mapped to trna, 0.13% of the reads mapped to mirna, 2.22% of the reads mapped to ENSEMBL_cdna and 11.16% of the reads mapped to ENSEMBL_ncrna. Figure 10 shows the heatmap with the corresponding significantly altered expression. A total of 89 significantly different miRNAs were found. Among these, one to 10 miRNAs were increased, and 44 to 68 miRNAs were decreased, depending on the treatment of the AdMSCs. In particular, chondrogenic differentiation under hypoxia showed notable results, where only one miRNA was upregulated (eca-miR-1307) and 68 miRNAs were expressed at low levels (Fig. 11). An overview of the significant changes in expression after the various treatment options compared with untreated AdMSCs is shown in Tables 1, 2, 3, 4 and 5 for chondrogenic differentiation (Table 1), chondrogenic differentiation under hypoxia (Table 2), treatment with IL-1β (Table 3), shock wave treatment (Table 4) and senescence (Table 5).

**Table 1:** List of genes whose expression is significantly changed after chondrogenic differentiation

Name	l2FC	Name	l2FC	Name	l2FC
eca-let-7a	3.79	eca-miR-16	4.6	eca-miR-28-5p	5.28
eca-let-7c	2.71	eca-miR-181a	2.88	eca-miR-29a	7.11
eca-let-7f	5.13	eca-miR-186	2.89	eca-miR-29b	4.32
eca-let-7 g	2.62	eca-miR-191a	2.85	eca-miR-30b	6.09
eca-miR-101	6.39	eca-miR-199a-3p	8.82	eca-miR-30d	3.56
eca-miR-106b	5.75	eca-miR-199a-5p	3.22	eca-miR-3613	4.54
eca-miR-10a	7.51	eca-miR-199b-3p	11.16	eca-miR-365	6.52
eca-miR-10b	9.22	eca-miR-19b	5.84	eca-miR-374a	6.49
eca-miR-125b-5p	2.49	eca-miR-21	8.18	eca-miR-423-5p	1.26
eca-miR-1271a	4.77	eca-miR-214	4.48	eca-miR-505	4.29
eca-miR-128	4.53	eca-miR-22	3.97	eca-miR-532-5p	3.62
eca-miR-143	7.07	eca-miR-23a	3.77	eca-miR-92a	1.3
eca-miR-145	5.55	eca-miR-23b	4.17	eca-miR-1307	-3.91
eca-miR-148a	6.3	eca-miR-24	2.34	eca-miR-744	-3.62
eca-miR-148b-3p	5.5	eca-miR-26a	3.65	eca-miR-8990	-4.65
eca-miR-15b	5.28	eca-miR-27a	3.25	eca-miR-99b	-2.04

The name of the miRNA (name) and the log2 fold change in gene expression (l2FC) compared with a negative control (untreated AdMSC). The p values are less than 0.05 and the exact values can be found in the supplement

**Table 2:** List of genes whose expression is significantly changed after chondrogenic differentiation under hypoxia

Name	l2FC	Name	l2FC	Name	l2FC
eca-let-7a	4.83	eca-miR-15a	5.15	eca-miR-25	2.91
eca-let-7c	4.29	eca-miR-15b	7.31	eca-miR-26a	5.02
eca-let-7d	4.65	eca-miR-16	6.28	eca-miR-28-3p	2.76
eca-let-7e	3.95	eca-miR-17	5.98	eca-miR-28-5p	6.2
eca-let-7f	6.24	eca-miR-181a	2.73	eca-miR-29a	7.06
eca-let-7 g	5.01	eca-miR-181b	3.56	eca-miR-29b	5.13
eca-miR-100	2.84	eca-miR-186	3.24	eca-miR-30b	5
eca-miR-101	7.04	eca-miR-18a	4.88	eca-miR-30d	3.4
eca-miR-103	6.45	eca-miR-196b	4.47	eca-miR-340-5p	5.47
eca-miR-106a	5.85	eca-miR-199a-3p	8.35	eca-miR-34a	4.97
eca-miR-106b	7.33	eca-miR-199a-5p	3.72	eca-miR-350	4.86
eca-miR-107a	5.25	eca-miR-199b-3p	10.75	eca-miR-365	6.8
eca-miR-10a	7.24	eca-miR-199b-5p	6.92	eca-miR-374a	8.23
eca-miR-10b	7.9	eca-miR-19a	5.87	eca-miR-424	5.78
eca-miR-125b-5p	4.22	eca-miR-19b	7.8	eca-miR-500	4.65
eca-miR-1271a	4.75	eca-miR-20a	6.55	eca-miR-503	6.15
eca-miR-143	6.89	eca-miR-21	8.17	eca-miR-505	4.24
eca-miR-145	3.4	eca-miR-214	2.71	eca-miR-532-5p	4.71
eca-miR-146b-5p	6.63	eca-miR-22	2.77	eca-miR-590-3p	4.82
eca-miR-148a	6.69	eca-miR-221	1.53	eca-miR-660	5.51
eca-miR-148b-3p	7.32	eca-miR-23a	4.39	eca-miR-93	4.89
eca-miR-151-5p	2.53	eca-miR-23b	3.28	eca-miR-98	5.68
eca-miR-155	4.11	eca-miR-24	3.13	eca-miR-1307	-4.2

The name of the miRNA (name) and the log2 fold change in gene expression (l2FC) compared with a negative control (untreated AdMSC). The p values are less than 0.05 and the exact values can be found in the supplement

**Table 3:** List of genes whose expression is significantly changed after IL-1b treatment

name	l2FC	name	l2FC	name	l2FC
eca-let-7a	2.91	eca-miR-16	5.6	eca-miR-29b	5.12
eca-let-7c	2.7	eca-miR-17	5.06	eca-miR-30b	3.85
eca-let-7d	2.54	eca-miR-186	1.97	eca-miR-30d	3.19
eca-let-7e	2.4	eca-miR-18a	4.41	eca-miR-340-5p	4.64
eca-let-7f	4.77	eca-miR-199a-3p	6.71	eca-miR-365	5.95
eca-let-7 g	3.89	eca-miR-199b-3p	9.06	eca-miR-374a	6.36
eca-miR-100	2.04	eca-miR-199b-5p	4.42	eca-miR-505	3.61
eca-miR-101	6.61	eca-miR-19a	5.56	eca-miR-532-5p	4.53
eca-miR-103	4.37	eca-miR-19b	7.05	eca-miR-660	5.09
eca-miR-106a	5.22	eca-miR-20a	5.75	eca-miR-8916	3.4
eca-miR-106b	6.71	eca-miR-21	6.9	eca-miR-8935	4.23
eca-miR-10a	6.78	eca-miR-214	1.74	eca-miR-92a	1.66
eca-miR-10b	7.43	eca-miR-22	2.11	eca-miR-93	3.91
eca-miR-125b-5p	3.61	eca-miR-221	2.03	eca-miR-98	4.8
eca-miR-128	4	eca-miR-23a	3.82	eca-miR-1307	-2.68
eca-miR-143	5.49	eca-miR-23b	3.01	eca-miR-328	-2.15
eca-miR-145	2.04	eca-miR-24	2.38	eca-miR-423-3p	-1.29
eca-miR-146a	6.71	eca-miR-25	2.3	eca-miR-423-5p	-1.21
eca-miR-146b-5p	5.65	eca-miR-26a	3	eca-miR-744	-2.3
eca-miR-148a	5.31	eca-miR-27a	2.62	eca-miR-8978	-2.15
eca-miR-148b-3p	5.5	eca-miR-28-5p	4.98	eca-miR-8990	-2.67
eca-miR-15a	3.62	eca-miR-29a	4.7	eca-miR-99b	-1.84
eca-miR-15b	6.51				

The name of the miRNA (name) and the log2 fold change in gene expression (l2FC) compared with a negative control (untreated AdMSC). The p values are less than 0.05 and the exact values can be found in the supplement

**Table 4:** List of genes whose expression is significantly changed after shockwave treatment

Name	l2FC	Name	l2FC	Name	l2FC
eca-let-7a	4.04	eca-miR-148a	4.65	eca-miR-28-5p	4.72
eca-let-7c	2.71	eca-miR-148b-3p	4.92	eca-miR-29a	4.81
eca-let-7d	3.46	eca-miR-15b	5.71	eca-miR-30b	3.5
eca-let-7e	3.24	eca-miR-16	4.65	eca-miR-340-5p	4.5
eca-let-7f	5.82	eca-miR-17	4.81	eca-miR-365	5.75
eca-let-7 g	3.66	eca-miR-199a-3p	6.41	eca-miR-374a	6.09
eca-miR-1	4.28	eca-miR-199b-3p	8.69	eca-miR-532-5p	3.41
eca-miR-100	1.9	eca-miR-199b-5p	5.36	eca-miR-660	4.53
eca-miR-101	5.57	eca-miR-19a	4.97	eca-miR-93	2.89
eca-miR-103	4.09	eca-miR-19b	6.43	eca-miR-1307	-2.56
eca-miR-106b	5.77	eca-miR-20a	4.71	eca-miR-328	-2.51
eca-miR-107a	3.72	eca-miR-21	6.53	eca-miR-423-3p	-1.48
eca-miR-10a	6.46	eca-miR-221	1.76	eca-miR-423-5p	-1.75
eca-miR-10b	6.87	eca-miR-23a	3.43	eca-miR-744	-2.04
eca-miR-125b-5p	3.78	eca-miR-23b	2.47	eca-miR-8928	-1.76
eca-miR-143	5.1	eca-miR-24	1.72	eca-miR-8978	-1.86
eca-miR-145	2.15	eca-miR-26a	3.27	eca-miR-8990	-3.56
eca-miR-146b-5p	4.31				

The name of the miRNA (name) and the log2 fold change in gene expression (l2FC) compared with a negative control (untreated AdMSC). The p values are less than 0.05 and the exact values can be found in the supplement

**Table 5:** List of genes whose expression is significantly changed in senescent equine AdMSCs

Name	l2FC	Name	l2FC	Name	l2FC
eca-let-7a	3.09	eca-miR-186	1.91	eca-miR-30b	3.74
eca-let-7c	2.39	eca-miR-188-5p	4.17	eca-miR-340-5p	4.53
eca-let-7d	3.17	eca-miR-196b	3.36	eca-miR-34a	4.9
eca-let-7f	4.6	eca-miR-199a-3p	6.74	eca-miR-365	6.03
eca-let-7 g	3.34	eca-miR-199a-5p	2.65	eca-miR-374a	5.97
eca-miR-100	1.91	eca-miR-199b-3p	9.11	eca-miR-424	5.16
eca-miR-101	5.61	eca-miR-199b-5p	5.87	eca-miR-503	5.08
eca-miR-103	5.08	eca-miR-19a	4.6	eca-miR-532-5p	4.02
eca-miR-106a	4.74	eca-miR-19b	6.44	eca-miR-660	5.27
eca-miR-106b	5.93	eca-miR-20a	5.16	eca-miR-93	3.2
eca-miR-107a	3.8	eca-miR-21	6.71	eca-miR-1307	-2.71
eca-miR-10a	6.21	eca-miR-214	1.5	eca-miR-193a-5p	-2.11
eca-miR-10b	7.41	eca-miR-221	1.54	eca-miR-197	-1.93
eca-miR-125b-5p	2.8	eca-miR-23a	3.56	eca-miR-328	-2
eca-miR-143	5.11	eca-miR-23b	3.14	eca-miR-423-3p	-1.7
eca-miR-145	2.77	eca-miR-24	2.54	eca-miR-423-5p	-1.58
eca-miR-148a	4.2	eca-miR-26a	3.35	eca-miR-744	-2.91
eca-miR-148b-3p	5.62	eca-miR-28-5p	5.65	eca-miR-8978	-2.44
eca-miR-15b	5.46	eca-miR-29a	6.42	eca-miR-8990	-4.39
eca-miR-16	4.37	eca-miR-29b	4.42	eca-miR-99b	-1.88
eca-miR-17	4.21				

The name of the miRNA (name) and the log2 fold change in gene expression (l2FC) compared with a negative control (untreated AdMSC). The p values are less than 0.05 and the exact values can be found in the supplement

## Discussion

Since MSCs are considered to act in part through the secretion of EVs, we focused on the characterization of miRNAs within these EVs. For this purpose, equine AdMSCs were stimulated or damaged in different ways in vitro. The change in the resulting gene expression in the EVs should provide information on their supportive action toward a healing process by finding potential candidates for miRNAs via overall analysis (next generation sequencing) of the miRNAs contained in the EVs.

The equine AdMSCs used for these studies have already been obtained and characterized in advance, which is why the methods were not described in detail. Nevertheless, the donors used in this study provided the evidence required by the International Society for Cellular Therapy (ISCT) standards [20]. In addition, proof of senescence was provided for more passages via beta-galactosidase staining. In stem cells as primary cell cultures, a passage above P3 is already considered senescent [33, 34], although there may be strong donor-dependent variations [35, 36].

The various treatment options for AdMSCs before harvesting EVs should, if possible, correspond to potential typical treatment modalities associated with OA as carried out in the equine clinic. In addition to a control of chondrogenic differentiation, equine AdMSCs were subjected to IL-1β stimulation, shock wave treatment and senescence. The aim was to reproduce different scenarios of the actual state or treatment:

### Chondrogenically differentiated AdMSCs under normoxia and hypoxia for the treatment of OA

1. IL-1β as a damage model for OA.
2. Shock wave as a treatment for OA.
3. Senescence as a possible cause or additional problem of the use of MSCs in the treatment of OA.

For chondrogenic differentiation, a monolayer culture was used instead of a 3D pellet culture. This was because no EVs could be obtained in significant quantities in the media supernatants of the pellet culture normally used for chondrogenic differentiation (data not shown). This ruled out this method for subsequent NGS analysis. Since hypoxic conditions prevail in the center of the pellets, instead of culturing the cells under normoxia, chondrogenic differentiation was performed in the monolayer under hypoxic culture conditions via an incubator with O_2_ regulation at an oxygen concentration of 3%. This was intended to replace the conditions of the pellet culture as much as possible.

After the cell culture supernatants of the treated equine AdMSCs were collected, the number and size distribution of the contained particles (including EVs) were measured via NTA for quality control. The size of the particles contained was found to be consistent with previous studies, regardless of pretreatment. The average EV size of 146 nm was within the expected range for EVs [24, 31, 32]. In contrast, the particle concentration was significantly more heterogeneous with 9.66 ± 5.39 × 108 (x̅ ± SD) particles/ml. The significantly higher number of particles after chondrogenic differentiation under hypoxia is a described phenomenon that can be attributed to hypoxia [33, 34].

Finally, the media supernatants were sent to a service provider for NGS. A total of 89 significantly altered miRNAs were found after different treatments. These were determined from different groups after treatment of AdMSCs. As seen previously in particle counts, most miRNAs were also found after chondrogenic differentiation with hypoxia, which was significantly different from the results of the negative control. We found a total of 69 significantly altered miRNAs, of which only miR-1307 was downregulated, and all other miRNAs were upregulated. The increased expression of miRNAs stimulated by hypoxia has been described previously, and here, we identified a specific set of miRNAs, the so-called hypoxemic, which are particularly highly expressed in cells stressed by hypoxia [35]. Many miRNAs were found to be up- or downregulated under hypoxia. However, compared with our results, some of the miRNAs expected to be downregulated, namely the miRNAs eca-let-7a/c/d/f, eca-miR-101, eca-miR-16, eca-miR-186, eca-miR-199a(-5p), eca-miR-20a and eca-miR-374(a), were actually upregulated. Among the more highly expressed miRNAs is the miRNA eca-miR-424. This miRNA is known to be a key miRNA in hypoxia, as it stabilizes hypoxia-inducible factor 1 (HIF-1) and thus supports the associated cell response to hypoxia [36].

Of greatest interest, however, are certainly those miRNAs that are thought to have a protective or rather deleterious effect in OA. A central idea was that AdMSCs are stimulated by pretreatment to secrete EVs with miRNA, which has a positive effect on damaged cells such as chondrocytes. However, it should be noted that the results reported in the literature are from a variety of fluids such as synovia [43, 44] and plasma [44, 45] or from different cells in vitro, such as from primary chondrocyte cultures [46] or synovial cell cultures with MSCs [47, 48]. A list of different protective and destructive miRNAs has already been published by several authors [49–53]. Notably, not only can an isolated miRNA be important, but protective and destructive miRNAs are also balanced in the healthy state [52].

Thus, within the category of anti-inflammatory miRNAs, miR-101 [37], miR-143 [38], mir-145 [18], miR-146a [39], miR-27a [40] and miR-93 [41] were identified. A comparison of these miRNAs revealed that eca-miR-101 was significantly increased under all conditions, and eca-miR-146a was significantly increased only in AdMSCs stimulated with IL-1β, a miRNA that is thought to attenuate IL-1β responses [39]. In addition to anti-inflammatory miRNAs, other protective miRNAs have identified, such as miR-98, which is supposed to prevent apoptosis of chondrocytes [42], and miR-29b(-5p), which induces chondrogenesis by preventing the senescence of chondrocytes [43]. The significantly elevated expression of miR-221 has also been shown to play a supportive role in chondrogenesis [18]. In a comprehensive analysis of blood serum and synovia from horse joints with induced OA, Anderson et al. 2022 were able to postulate further miRNAs important for OA with the help of an Ingenuity Pathway Analysis, of which we were able to find a total of 23 miRNAs in our investigations, one of which has already been described here (eca-miR-93), and the following [44]: eca-let-7a, eca-let-7c, eca-miR-103, eca-miR-107a, eca-miR-10a, eca-miR-10b, eca-miR-128, eca-miR-16, eca-miR-199a-3p, eca-miR-199b-3p, eca-miR-19b, eca-miR-21, eca-miR-23a, eca-miR-25, eca-miR-26a, eca-miR-28-5p, eca-miR-30d, eca-miR-423-3p, eca-miR-532-5p, eca-miR-744, eca-miR-92a and eca-miR-99b.

In addition to the numerous miRNAs whose expression increased after the various AdMSC treatments, one miRNA, namely eca-miR-1307, was also significantly reduced in all the samples. This miRNA has often been described as a tumor marker that supports the growth of tumors and can also induce resistance to chemotherapeutic agents [45, 46]. This is thought to occur by blocking apoptosis and improving DNA repair [47]. Why this miRNA in particular showed reduced gene expression in all samples cannot be interpreted without further investigations.

In summary, many miRNAs, whose effects are still unknown or have not been sufficiently investigated, have been identified by our studies specifically and exclusively in horses. At this point, we would also like to mention the small number of animals examined for NGS. It was not possible to include more donors in this study due to the high effort involved. We tried to compensate for this as much as possible by selecting three heterogeneous donors. Due to the comparatively high number of significantly altered miRNA values, we assume that the results provide a good overview for the equine species. The applicability of the results from the equine to other species, such as other domestic mammals or primates including humans, cannot be conclusively guaranteed. However, horses can be considered model animals for musculoskeletal diseases [48, 49]. In addition, 140 equine miRNAs that are 100% identical to human miRNAs have already been identified [50]. Among these homologous miRNAs, we were able to find a total of 41 different miRNAs in our studies (Table 6).

**Table 6:** AdMSC-derived miRNAs from equine AdMSCs after various stimuli, which show 100% homology to human MiRNAs

Equine miRNAs with 100% homology to human miRNAs				
eca-let-7d	eca-miR-148b-3p	eca-miR-197	eca-miR-221	eca-miR-423-3p
eca-let-7e	eca-miR-151-5p	eca-miR-199a-5p	eca-miR-23a	eca-miR-423-5p
eca-miR-103	eca-miR-15b	eca-miR-199b-3p	eca-miR-27a	eca-miR-424
eca-miR-106b	eca-miR-186	eca-miR-199b-5p	eca-miR-28-3p	eca-miR-500
eca-miR-125b-5p	eca-miR-188-5p	eca-miR-19a	eca-miR-28-5p	eca-miR-505
eca-miR-128	eca-miR-18a	eca-miR-19b	eca-miR-29a	eca-miR-92a
eca-miR-145	eca-miR-191a	eca-miR-21	eca-miR-29b	eca-miR-93
eca-miR-146a	eca-miR-196b	eca-miR-214	eca-miR-328	eca-miR-99b
eca-miR-148a				

Thus, with respect to the altered gene expression of miRNAs, it is not possible to define in general terms which miRNAs would be potentially beneficial in an OA treatment without further in vivo studies. It can be argued that unstressed AdMSCs may already produce a potent reservoir of secreted EVs, but after these treatments, their potential seems to be greatly increased. To take this into account, great attention should be given to the expression profiles after different treatments, as these profiles provide information about the conditions that may prevail in vivo in a disease. Therefore, it would be conceivable to eliminate unwanted miRNAs specifically at the site of action. This could be accomplished either with suitable, possibly synthetically produced miRNA carriers, which are protective miRNAs, or directly with anti-miRNAs [51].

## Conclusion

With our studies on the EVs of equine AdMSCs after different types of stimulation, we were able to find significant changes in the expression of several miRNAs, which should be further investigated in the near future. These findings provide a valuable basis for the search for corresponding miRNAs for therapeutic purposes in humans and animals or for further deciphering the function of AdMSCs.

## Electronic supplementary material

Below is the link to the electronic supplementary material.

Supplementary Material 1

Supplementary Material 2

Supplementary Material 3

Supplementary Material 4

Supplementary Material 5

## Data Availability

All data generated or analyzed during this study are included in this published article and its supplementary information files.

## Declarations

### Artificial intelligence (AI)

The authors declare that they have not use AI-generated work in this manuscript.

## Abbreviations

AdMSC: Adipose derived mesenchymal stem cells
ISCT: International Society for Cellular Therapy
ITS: Insulin-transferrin-selenite
NGS: Next Generation Sequencing
OA: Osteoarthritis
SD: Standard derivation
